# Burnout and Cognitive Load Among Primary Care Clinicians: A Cross-Sectional Mixed-Methods Survey Study

**DOI:** 10.21203/rs.3.rs-8929231/v1

**Published:** 2026-04-07

**Authors:** Yohali Burrola-Mendez, Imara I. West, Maria G. Prado, Tracy M. Anastas, Christopher W. Lewis, Angad P. Singh, Matthew B. Jaffy, Kari A. Stephens

**Affiliations:** University of Washington; University of Washington; University of Washington; University of Washington; University of Washington; University of Washington; University of Washington; University of Washington

**Keywords:** Burnout, Professional, Primary Health Care, Cognitive Load, Workload, Electronic Health Records

## Abstract

**Background:**

Burnout is highly prevalent among primary care clinicians (PCCs) and is associated with reduced job satisfaction, clinician turnover, and lower quality of care. While administrative burden and electronic health record (EHR) complexity are recognized contributors, less attention has been paid to cognitive load, the mental effort required to process and act on complex clinical information, as a potential mechanism underlying burnout. Cognitive load theory suggests that excessive mental and temporal demands may impair performance and increase strain, yet few studies have examined cognitive load and burnout concurrently in primary care. We aimed to assess the prevalence of burnout and cognitive load among PCCs, evaluate differences by clinician characteristics, and examine associations between cognitive load domains and burnout.

**Methods:**

We conducted a cross-sectional survey of PCCs within the University of Washington Medicine Primary Care healthcare system (August–December 2023). Burnout was measured using the 9-item Abbreviated Maslach Burnout Inventory (aMBI) and defined as high emotional exhaustion (>8) and/or depersonalization (>5). Cognitive load was assessed using the NASA Task Load Index (NASA-TLX). Linear regression models examined associations between burnout status and NASA-TLX domains, adjusting for age, gender, race, ethnicity, percentage of time performing clinical work, and years of clinical practice. Open-ended responses were analyzed inductively using thematic analysis.

**Results:**

Among 68 clinicians with complete data (mean age 44.4 years; 73.5% non-male), 77.9% met criteria for burnout. PCCs with burnout reported significantly higher mental demand (*β* = 14.79, *p* = 0.01), temporal demand (*β* = 18.93, *p* = 0.01), and frustration (*β*= 34.59, *p* < 0.001) compared with those without burnout. Qualitative findings identified five themes: administrative overload and EHR complexity; time pressure and heavy workload; structural system gaps; diminished relational and diagnostic quality; and emotional exhaustion and moral injury. Clinicians with high burnout emphasized emotional depletion and systemic frustration.

**Conclusions:**

Burnout among primary care clinicians was common and was associated with higher reported mental and temporal demands and frustration. These findings suggest that cognitive load and burnout are closely related within contemporary primary care environments characterized by high administrative and clinical complexity. Interventions aimed at optimizing workflows and reducing unnecessary cognitive burden warrant further investigation to determine whether addressing cognitive load may contribute to improved clinician well-being.

## Background

Burnout is a complex, chronic state characterized by emotional exhaustion, depersonalization, and reduced personal accomplishment, resulting from prolonged stress that is not effectively managed (1, 2). Primary care clinicians (PCCs) commonly express burnout, which is associated with lower job satisfaction, higher clinician turnover, and lower quality of patient care and satisfaction (3). In the United States, prevalence estimates across all PCC specialties range from 13.5% to 60% (3) and across primary care physicians from 42%–51% (4), with the primary care practice environment identified as the most consistent predictor (3).

Cognitive load is another important, but less frequently examined dimension of clinician work stress that specifically refers to the mental effort required to process, prioritize, and act on complex information during clinical care (5, 6). In primary care, high cognitive load has been associated with documentation and usability burdens in the electronic health records (EHR) and with complex patient care demands (7, 8). In experimental and simulation studies, elevated cognitive load has been linked to higher electronic prescribing error rates and automation bias (9) and to biased treatment decisions in chronic pain care (10).

Despite the well-documented prevalence of burnout and the recognized influence of cognitive load on clinical performance, few studies have examined both constructs concurrently among PCCs and their association. Understanding how cognitive load contributes to burnout and affects professional experiences is essential for developing interventions that optimize workflows, manage workload, and support clinician well-being to address the increasing trend of primary care workforce burnout (11) and the worsening primary care shortage (12).

The primary aim of this study was to assess levels of burnout and cognitive load among PCCs, examine differences by demographic and professional characteristics, and evaluate the association between cognitive load and burnout. A secondary aim was to explore clinicians’ perceptions of how cognitive load affects their work through analysis of open-ended survey responses to better understand how these constructs intersect in primary care practice. We hypothesized that clinicians reporting higher levels of burnout would also report higher cognitive load.

## Methods

### Study Design and Setting

We conducted a cross-sectional survey study of PCCs at the University of Washington (UW) Medicine Primary Care healthcare system from August to December 2023. The aim of this study was to examine the prevalence of burnout and cognitive load and to evaluate associations between cognitive load domains and burnout status. The UW Institutional Review Board determined the study exempt from human subjects research oversight (Study00017123).

### Participants and Recruitment

Eligible PCCs were attending physicians, residents/fellows, physician assistants, and nurse practitioners currently providing primary care and had provided primary services at UW for at least 6 months. PCCs were recruited via institutional email using convenience sampling and were invited to complete an online survey.

A total of 182 eligible clinicians were invited to participate. Electronic informed consent was obtained. Surveys were de-identified using randomly generated usernames, and participants received a $15 gift card upon completion of the survey.

### Measures

The survey included four sections:

#### Demographics.

The questionnaire was developed by the research team. It included demographic variables (age, gender, race, and ethnicity) and clinical/work variables (clinician type, percent of time spent in clinical work, and years in clinical practice) (see Appendix 1, Section A).

#### Burnout.

Burnout was assessed using the 9-item abbreviated Maslach Burnout Inventory (aMBI), rated on a 7-point Likert scale (0 = never to 6 = daily). The instrument measures Emotional Exhaustion (EE), Depersonalization (DP), and Personal Accomplishment (PA). Higher EE and DP scores indicate greater burnout, while higher PA scores indicate lower burnout. High burnout was defined a priori as EE > 8 and/or DP > 5, consistent with established thresholds. Burnout was operationalized as a binary variable (high vs. low symptoms) for regression analyses. The aMBI has demonstrated acceptable reliability and validity in healthcare populations (13, 14).

#### Cognitive workload.

Cognitive load was assessed using the NASA Task Load Index (NASA-TLX), which measures six domains: mental demand, physical demand, temporal demand, performance, effort, and frustration; each scored from 0 (very low) to 100 (very high). Participants were instructed: “*Thinking about the past month, please answer the following questions regarding your experience while performing clinical work (e.g., interacting with patients, charting) in outpatient primary care or urgent care settings.*” The NASA-TLX has demonstrated strong reliability and validity in adult population (15).

#### Perceptions of cognitive load.

Six Likert-type items were developed by the research team to assess PCC perceived cognitive load in clinical care, including typical cognitive load, perceived ability to manage it, effects of caring for patients with chronic pain or opioids, the impact of opioid-prescribing guidelines, and perceived consequences of high cognitive load. Two open-ended questions asked about additional impacts on care and cognitive load or burnout experience (see Appendix 1, section B). Survey responses were collected electronically using the Research Electronic Data Capture (REDCap)(16).

### Analyses

#### Quantitative Analysis.

Descriptive statistics summarized provider demographic and clinical characteristics, aMBI domain scores, burnout symptoms, NASA-TLX cognitive load scores, and other survey responses. Continuous variables were summarized as means and standard deviations, and categorical variables as frequencies and percentages.

Unadjusted comparisons of provider characteristics by burnout symptom status were assessed using Fisher’s exact tests for categorical variables and *t*-tests for continuous variables.

Multivariable linear regression models were fit separately for each NASA-TLX domain as the dependent variable. Burnout status (high vs. low) was the primary independent variable. Models adjusted for age, gender, race, ethnicity, percentage of time performing clinical work, and years of clinical practice.

Regression coefficients (β) and standard errors were reported. Statistical significance was defined as p < 0.05. Analyses were conducted using SAS 9.4 (SAS Institute Inc.). Complete-case analysis was used.

Given the exploratory nature of this study and the fixed size of the clinician population within the health system, no formal a priori power calculation was performed.

#### Qualitative analysis.

Open-ended survey responses were analyzed using an inductive thematic analysis approach. Two researchers independently coded responses, met to reconcile discrepancies, and iteratively developed a codebook and higher-order themes. Themes were examined by burnout status to identify differences in emphasis or framing. Representative quotations were selected to illustrate key patterns. Analyses followed principles of applied thematic analysis (17).

## Results

A total of 182 eligible clinicians were invited to participate. Seventy-eight PCCs (42.8%) completed the survey. Of these, 68 (37.4% of those invited) had complete demographic, aMBI, and NASA-TLX data and were included in the analytic sample.

PCCs had a mean age of 44.4 years (SD 11.9); 73.5% were non-male, and 76.5% identified as White. Most were physicians (70.6%), with the remainder being Advanced Practice Practitioners (29.4%). The majority of PCCs reported spending at least half of their FTE performing (69.1%) clinical work and had practiced for 0–10 years (51.5%).

Overall, 77.9% of providers met criteria for burnout (EE > 8 or DP > 5). NASA-TLX scores were highest for mental demand (*M* = 78.7, *SD* = 16.6), effort (*M* = 76.1, *SD* = 19.5), and temporal demand (*M* = 73.0, *SD* = 19.0). Full descriptive statistics are presented in Table 1.

### Clinicians Characteristics by Burnout Status

PCCs with high levels of burnout were more likely to be non-male (86.0% vs. 44.4%, *p* = 0.02) and had fewer years in practice (*p* = 0.04). No significant differences were observed by age, race, ethnicity, clinician type, or percentage of time spent in clinical care (Table 2).

### Associations between Burnout and Cognitive Load

In multivariable linear regression analyses, burnout status was significantly associated with several domains of cognitive workload. After adjusting for age, gender, race, ethnicity, percentage of time performing clinical work, and years of clinical practice, clinicians with high burnout had higher NASA-TLX scores for mental demand (*β* = 14.79, *SE* = 5.56, *p* = 0.01), temporal demand (*β* = 18.93, *SE* = 6.62, *p* = 0.01), and frustration (*β* = 34.59, *SE* = 8.61, *p* < 0.001) compared with those with low burnout. No significant differences were observed for physical demand, effort, or perceived performance (Table 3).

### Clinicians’ perceptions of burnout and clinical cognitive load

Most PCCs rated their average cognitive load as *high* (45.6%) or very *high* (44.1%) during clinical duties. While over two-thirds (67.6%) reported managing their cognitive load *fairly* or *well*, those with high burnout were less likely to be managing it *very well* compared with those with low burnout (*p* = 0.03). Nearly all indicated that caring for patients with chronic pain (82.3%) or those prescribed opioids (76.5%) increased cognitive load, with no differences by burnout status. When asked about consequences of high cognitive load, 50% identified clinician burnout as the primary consequence, followed by less patient-centered care (27.9%) and medical errors (10.3%) (see Appendix 2).

Of 68 total PCCs included in the analytic sample, 35 (51%) provided written responses to first open-ended question, “*If there are other ways you think clinician cognitive load has a significant impact on patient care that are not listed above, please describe them here*”; and 30 (44%) to second “*If you have any additional comments about clinician cognitive load or burnout, please include them here*”. Inductive thematic analysis identified five themes describing how cognitive load shapes experiences in primary care. Themes were observed across burnout groups; however, clinicians with high burnout more frequently emphasized emotional exhaustion, system frustration, and moral distress, whereas those with low burnout more often described discrete and potentially modifiable barriers.

#### 1. Administrative overload and EHR complexity limit clinical care

Across groups, PCCs described heavy EHR and administrative demands, such as inbox management, documentation, billing, and redundant communication, that displaced patient care. Inefficiency and constant interruptions were cited as major sources of cognitive strain.

##### High levels of burnout:

- “For every eight hours in clinic, I spend an extra three to four hours on charting and inbox management before bed. It’s taking a toll on my family and makes me question staying in primary care.”
- “I spend hours in Epic just figuring out how to do what I need or documenting things already done. It’s wasting time I could use helping people.”

##### Low levels of burnout:

- “High cognitive load can be a good thing; I just shouldn’t have to have high cognitive load using the EHR.”

#### 2. Time pressure and heavy workload lead to shortcuts and compromise care quality

PCCs described insufficient time to manage clinical and administrative responsibilities, leading to deferred issues, increased referrals, or unnecessary testing.

##### High levels of burnout:

- “It’s really impossible to do everything in a day. Between visits, coordination, MyChart, and inbox, care suffers because there’s just not enough time.”
- “High cognitive load in short visits causes deficiencies: we refer more, limit hearing concerns, and order imaging because it’s faster.”

##### Low levels of burnout:

- “As an internist, I expect to have high cognitive load.”

#### 3. Structural gaps: inadequate staffing, appointment access, training, and shifting initiatives

PCCs attributed cognitive strain to system-level factors including inadequate staffing, limited appointment availability, fragmented onboarding, and constantly shifting initiatives.

#### High levels of burnout:

- “Too many new requirements and projects each week, keeping up with them adds to the load of every visit.”
- “This system is completely broken. Colleagues cut their FTEs to cope, but panels and expectations stay the same.”

#### Low levels of burnout:

- “The biggest obstacles are lack of staff and appointment slots, figuring out how to handle a patient’s work-up when there are no appointments for months.”

### 4. Diminished relational and diagnostic quality

High cognitive demands reduced clinicians’ emotional presence and ability to listen, affecting therapeutic relationships and diagnostic accuracy. They described less empathy, rushed encounters, and declining trust.

#### High levels of burnout:

- “Depersonalized care and worsening relationships, patients feel unvalued or that their concerns aren’t worth our time.”
- “All the paperwork and messages destroy trust between patient and provider.”

#### Low levels of burnout:

- “I spend less time listening and am less able to pick up important cues.”

### 5. Emotional exhaustion, moral injury, and intent to leave

PCCs with high burnout described emotional depletion, guilt over compromised care, and moral injury, with many considering reducing hours or leaving practice.

#### High levels of burnout:

- “This isn’t burnout—it’s abuse. The expectations are impossible, and it’s breaking people.”
- “I try to be the doctor I trained to be, but time pressure makes that impossible. We’re failing our patients and feel responsible.”

#### Low levels of burnout:

- *“It affects how present and empathic I can be with patients. Adding inbox and pharmacy issues takes a toll.*”

## Discussion

In this cross-sectional survey of PCCs, burnout was highly prevalent, affecting over three-quarters of respondents. Clinicians with high burnout reported significantly higher mental and temporal demands and greater frustration compared with those without burnout, even after adjusting for demographic and professional characteristics. Qualitative findings reinforced these quantitative associations, highlighting administrative burden, time pressure, and structural inefficiencies as central contributors to cognitive strain and emotional exhaustion. Together, these findings suggest that cognitive load and burnout are closely intertwined within contemporary primary care practice.

Consistent with prior literature, clinicians identified EHR-related tasks and administrative burden as dominant sources of cognitive overload and frustration (7). Respondents described spending additional hours outside of clinic managing inbox messages, documentation, billing requirements, and redundant communication, often at the expense of personal time and family relationships. These narratives align with studies linking clerical burden and insufficient documentation time to higher odds of burnout (18). Our findings extend this literature by demonstrating that specific domains of cognitive workload, particularly mental and temporal demands, are elevated among clinicians reporting burnout.

The relationship between cognitive load and burnout is an emerging area in clinician well-being research. A national study reported a strong association between task load and burnout independent of demographic and practice characteristics (19). Similarly, in our study, elevated mental and temporal demands were independently associated with burnout status. Qualitative responses described daily pressures that constrained thorough assessment, led to deferral of complex issues, and contributed to frustration. These findings suggest that chronic cognitive strain may represent one dimension of the broader work environment associated with burnout.

Early-career PCCs were more likely to experience burnout, consistent with prior research identifying increased vulnerability during the initial years of practice (20, 21). Multiple factors may contribute, including transition stress, workload expectations, reduced clinical autonomy, and the challenges of adapting to complex administrative systems. These findings suggest that clinicians in earlier career stages may experience heightened strain within demanding practice environments. Addressing this pattern may require both system-level approaches, such as workflow redesign, team-based care, and improved administrative support, and individual-level supports, including mentorship, training in managing cognitive demands, and protected time for professional development. Further research is needed to evaluate which strategies are most effective in mitigating burnout during early career stages.

Burnout was more common among non-male clinicians, consistent with prior research studies (22). Multiple factors have been proposed to explain these disparities, including compensation inequities, workplace cultural expectations, and differences in emotional and communication labor (22). Prior studies suggest that non-male clinicians often engage in more patient-centered communication and may receive higher volumes of electronic messages, which could contribute to increased cognitive and administrative workload (23, 24). While our study was not designed to examine mechanisms underlying these differences, the findings underscore the importance of considering how workload distribution and administrative demands may differentially affect clinicians across gender identities.

Advances in technology, including artificial intelligence (AI)–assisted documentation tools, may influence clinician workload. While early evidence suggests potential reductions in documentation burden (25, 26), their impact on cognitive load and burnout remains uncertain. Longitudinal and experimental studies are needed to determine whether such tools meaningfully alter clinician workload or well-being.

## Limitations

This study has several limitations. The sample was drawn from a single academic health system and lacked racial diversity, which may limit generalizability. The response rate was modest, and participation may have been influenced by clinician workload or interest in burnout, introducing potential response bias. The cross-sectional design precludes inference about directionality or causality between cognitive load and burnout. Comparisons between clinicians with high and low burnout should be interpreted cautiously given the small size of the low-burnout group. Open-ended survey responses were brief and may not capture the full depth of clinicians’ experiences. Additionally, cognitive load and burnout were assessed via self-report, which may be subject to reporting bias. Despite these limitations, the integration of quantitative and qualitative data offers complementary perspectives on how cognitive load and burnout are experienced in primary care. The mixed-methods approach strengthens interpretation of the findings by contextualizing statistical associations within clinicians’ lived experiences.

## Conclusion

Burnout and cognitive load were strongly associated among primary care clinicians in this health system. Elevated mental and temporal demands and frustration characterized clinicians reporting burnout, underscoring the cognitively intensive nature of contemporary primary care practice. These findings highlight the importance of understanding cognitive workload within clinical environments and suggest that efforts to optimize workflows and reduce unnecessary cognitive burden warrant further study. As digital tools, including AI-assisted documentation, continue to evolve, longitudinal research is needed to determine their impact on clinician workload, well-being, and care quality.

## Supplementary Material

Supplementary Files

This is a list of supplementary files associated with this preprint. Click to download.

• Appendix.docx

## Figures and Tables

**Table 1.** Primary Care Clinicians Characteristics, Burnout, and Cognitive Workload (*N* = 68)

Characteristics	*N (%)*

**Gender identity**	
Male	18 (26.5)
Female	49 (98)
Nonbinary/fluid/gender queer	1 (2)
**Race**	
White	52 (76.5)
Non-White	16 (23.5)
American Indian or Alaskan Native	1 (6.2)
Asian	13 (81.2)
More than One Race	2 (12.5)
**Ethnicity**	
Hispanic or Latino	3 (4.4)
Not Hispanic or Latino	65 (95.6)
**Clinician Type**	
Physicians	48 (70.6)
Attending physicians	45 (66.2)
Residents	3 (4.4)
Advanced Practice Practitioners	20 (29.4)
Nurse practitioners	17 (25)
Physician assistants	3 (4.4)
**Time Spent Performing Clinical Work (%)**	
0–49	21 (30.9)
50–100	47 (69.1)
**Years Practicing**	
0–10	35 (51.5)
11–20	15 (22.1)
>20	18 (26.5)
**Burnout Status**	
High Symptoms of Burnout	53 (77.9)
Low (no) Symptoms of Burnout	15 (22.1)
	
	**Mean (SD)**
**Age**	44.4 (11.9)
**aMBI Domain Scores**	
Emotional Exhaustion	10.9 (4.5)
Depersonalization	4.6 (3.7)
Personal Accomplishment	15.0 (2.6)
**aMBI Domain Scores by Burnout Status**	
High Symptoms of Burnout	
Emotional Exhaustion	12.5 (3.5)
Depersonalization	5.5 (3.6)
Personal Accomplishment	14.8 (2.8)
Low (no) Symptoms of Burnout	
Emotional Exhaustion	5.1 (2.0)
Depersonalization	1.3 (1.4)
Personal Accomplishment	15.7 (1.5)
**NASA-TLX Subscale Scores**	
Mental Demand	78.7 (16.6)
Physical Demand	31.4 (25.4)
Temporal Demand	73.0 (19.0)
Performance	35.1 (19.9)
Effort	76.1 (19.5)
Frustration Level	56.4 (26.4)

*Legend.* Percentages for race subcategories are calculated among non-White participants. SD = standard deviation.

**Table 2.** Participant Characteristics by Burnout Status Among Primary Care Providers (*N* = 68)

Characteristic	Total (*n*)	High Symptoms of Burnout N=53	Low Symptoms of Burnout N=15	*p*-value*
**Age, years, mean (SD)**	44.4 (11.9)	43.2 (10.7)	48.4 (15.1)	0.14
**Gender (*n*, %)**				**0.02**
Male	18	10 (55.6)	8 (44.4)	
Non-male	50	43 (86.0)	7 (14.0)	
**Race (*n*, %)**				0.74
White	52	41 (78.8)	11 (21.2)	
Non-White	16	12 (75.0)	4 (25.0)	
**Ethnicity (*n*, %)**				0.12
Hispanic or Latino	3	1 (33.3)	2 (66.7)	
Not Hispanic or Latino	65	52 (80.0)	13 (20.0)	
**Clinician Type (*n*, %)**				0.35
MD/DO	48	39 (81.3)	9 (18.8)	
Attending physicians	45	36 (80.0)	9 (20.0)	
Residents	3	3 (100.0)	0 (0.0)	
NP/PA	20	14 (70.0)	6 (30.0)	
Nurse practitioners	17	13 (76.5)	4 (23.5)	
Physician assistants	3	1 (33.3)	2 (66.7)	
**Time Spent Performing ClinicaiWork (*n*, %)**				0.20
0–49	21	14 (66.7)	7 (33.3)	
50–100	47	39 (83.0)	8 (17.0)	
**Years Practicing (*n*, %)**				**0.04**
0–10	35	30 (85.7)	5 (14.3)	
11–20	15	13 (86.7)	2 (13.3)	
>20	18	10 (55.6)	8 (44.4)	

*Legend*.

* p value based on Fisher’s Exact test or t-test; significance set at *p* < 0.05.

**Table 3.** Clinicians’ NASA-TLX Cognitive Workload Scores by Burnout Status (*N* = 68)

NASA-TLX Subscale	High Symptoms of Burnout, Mean (*SD*), *n* = 53	Low Symptoms of Burnout, Mean (*SD*), *n* = 15	*β* [*SE*]	*t*	*p*-value
Mental Demand	80.9 (15.0)	70.7 (19.7)	14.79 [5.56]	2.66	**0.01**
Physical Demand	30.5 (25.1)	34.5 (27.0)	−4.19 [9.08]	−0.46	0.65
Temporal Demand	75.7 (16.8)	63.3 (23.2)	18.93 [6.62]	2.86	**0.01**
Performance	37.2 (19.8)	27.9 (19.3)	9.49 [7.35]	1.29	0.20
Effort	77.1 (19.5)	72.5 (19.4)	4.34 [6.84]	0.63	0.53
Frustration Level	63.4 (22.5)	31.3 (24.5)	34.59 [8.61]	4.02	**<0.001**

*Legend*. Models adjusted for age, gender, race, ethnicity, percentage of clinical effort, and years inpractice. β indicates the estimated difference in NASA-TLX score between providers with high and lowburnout. SE = standard error. SD = standard deviation.

## Data Availability

The datasets generated and/or analyzed during the current study are not publicly available due to the potential identifiability of participants within a single health system but are available from the corresponding author on reasonable request and with appropriate institutional approvals.

## Abbreviations

aMBI: Abbreviated Maslach Burnout Inventory
NASA-TLX: NASA Task Load Index
PCC: Primary Care Clinician
EHR: Electronic Health Record
